# Estimating the effect of optimizing anticancer drug vials on medical costs in Japan based on the data from a cancer hospital

**DOI:** 10.1186/s12913-020-05822-1

**Published:** 2020-11-09

**Authors:** Koichi Matsuo, Hisanaga Nomura, Masanobu Uchiyama, Motoyasu Miyazaki, Osamu Imakyure

**Affiliations:** 1 Department of Pharmacy, Fukuoka University Chikushi Hospital, Fukuoka, 818-8502 Japan; 2 Department of Pharmaceutical and Health Care Management, Faculty of Pharmaceutical Sciences, Fukuoka University, Fukuoka, Japan; 3 Department of Pharmacy, National Cancer Center Hospital East, Chiba, Japan

**Keywords:** Drug vial optimization (DVO), Reduction cost, Vial-sharing, Drug waste, Anticancer drug

## Abstract

**Background:**

The substantial increase in the use of expensive anticancer drugs has been accompanied by an increase in the amount of disposing residual liquid from drug preparations. Many Western countries, including the United States, have implemented drug vial optimization (DVO) to prevent the waste of anticancer drugs and have reported the reductions in the total drug costs. This study was designed to estimate the expected reduction in spending on anticancer drugs by Japanese cancer hospitals when DVO was implemented instead of individual preparations and to test the effectiveness of this approach.

**Methods:**

We investigated the doses of drugs used and quantity specifications for individually prepared vials for patients who received anticancer drug treatment in December 2017 at the Outpatient Treatment Center of the National Cancer Center Hospital East. Based on these findings, we calculated the total quantity of each drug used on a given day, and the minimum cost for preparation of the number of specified combinations corresponding to the total cost (DVO preparation). Based on the differences in these two costs, we estimated the economic impact of implementing DVO.

**Results:**

While the cost for anticancer drugs for the 1-month study period was US$3,305,595 (US$1 = \110) for individual preparations, the estimated cost for DVO preparations was US$3,092,955, equivalent to a reduction of US$212,640.

**Conclusions:**

Based on these study results, implementation of DVO-based preparation of injectable anticancer drugs in Japan in 2017 would have resulted in saving approximately US$460 million. This calculation revealed the need for the Japanese government to modify the methods employed to calculate drug costs in the insurance system and develop policies for the proper and optimal use of medical resources.

## Background

Japan’s anticancer drug market exceeded US$10 billion in 2017, and this value is expected to increase to approximately US$13 billion in 2020 [1]. The significant increase in the use of expensive anticancer drugs has been accompanied by an increase in the amount of residual liquid discarded from drug preparations [2–5]. Under the Japanese insurance system, the final medical cost is calculated as the total amount of vial usage for each drug used for each patient. For this reason, the Guidance on Safe Multiple Use of Injectable Anticancer Drugs was issued by Japan’s Ministry of Health, Labour and Welfare in 2018. However, the current medical insurance system provides little financial incentive for medical institutions to implement drug vial optimization (DVO). Consequently, drug waste accumulates, leading to unnecessary medical spending. In Japan and other countries, reducing unnecessary costs associated with the disposal of anticancer drugs is a key factor in the optimization of medical resources. By contrast, many Western countries, including the United States, have implemented DVO to increase the efficiency of anticancer treatment, resulting in decreases in the total drug costs [6–9]. However, because of the aforementioned reasons, there has been little progress in reducing drug waste in Japan. Maintaining the current method of disposing large amounts of expensive anticancer drugs in Japan is inefficient in terms of medical economics. Therefore, this study aimed to estimate the potential cost savings for the implementation of DVO in cancer hospitals in Japan. To the best of our knowledge, analyses of this issue have rarely been reported.

## Methods

### Aim

The aim of this study was to estimate cost savings that could be realized with the implementation of DVO preparation in cancer hospitals in Japan.

### Design and setting of the study

We investigated treatment regimens, drug doses, and quantities of drugs using vial specifications for individual patients who received anticancer treatment in December 2017 at the Outpatient Treatment Center of the National Cancer Center Hospital East. These data were generated using the anonymized per-patient doses of anticancer drugs extracted from the hospital electronic medical records and imputed in the regimen management system. The amount calculated using this method represented the total level of vial usage of each drug used in Japan, in where DVO had not been implemented. We then used these data to calculate the total quantities and costs of each drug used on a daily basis along with the minimum cost for specified individual combinations of drugs prepared using DVO. The amount calculated using this method represented the level of drug consumption when DVO is implemented and the minimum amount is reached. The difference between in these two costs reflected the estimated cost reduction expected from the implementation of DVO.

### The characteristics of the participants

All patients treated at the Outpatient Treatment Center of National Cancer Center Hospital East during the survey period were included in this study.

### Statistical analysis

Microsoft Office Excel 2016 (Microsoft, Redmond, WA, USA) was used to compile and analyze the data in this study; however, no statistical analysis was performed.

### Ethics approval

Because this report only examined the quantities of anticancer drugs used and thus, the study was a non-medical study that did not require an ethical review or approval under “Ethical Guidelines for Medical and Health Research Involving Human Subjects” in Japan.

## Results

The investigation involved 7 diagnostic departments, 2354 patients, and 3346 drugs. Table 1 presents a summary of the quantity of the top 5 most used drugs.

**Table 1:** The summary of the quantity of the top 5 most used anticancer drugs by medical department in the study period

		Gastroenterology (stomach, esophagus, colonm, etc.)	Number of cases		Hepatobiliary and pancreatic medicine (liver, gallbladder, pancreas, etc.)	Number of cases		Breast and medical oncology (breast, kidneys, prostate, etc.)	Number of cases		Respiratory medicine (lungs, mesothelioma, etc.)	Number of cases		Head and neck medicine (thyroid, tongue, etc.)	Number of cases		Hematology and oncology (lymphoma, myeloma, etc.)	Number of cases
Top 5	1	Fluorouracil	465	1	Gemcitabine	378	1	Trastuzumab	129	1	Nivolumab	62	1	Cetuximab	73	1	Rituximab	11
	2	Bevacizumab	262	2	nab-Paclitaxel	232	2	Paclitaxel	102	2	Pemetrexed	34	2	Nivolumab	60	2	Azacitidine	10
	3	Oxaliplatin	260	3	Cisplatin	65	3	Cyclophosphamide	38	3	Pembrolizumab	32	3	Paclitaxel	47	3	Bortezomib	8
	4	Irinotecan	171	4	Irinotecan	48	4	(3rd) Eribulin	38	4	Amrubicin	30	4	Carboplatin	26	4	Bendamustine	6
	5	Paclitaxel	71	5	Fluorouracil	46	5	Doxorubicin	30	5	Carboplatin	18	5			4	Doxorubicin	6
							5	Pertuzumab	30									

The total number of vials used for each drug was then utilized to estimate the vial usage of each drug under the assumption that DVO was implemented in the manner described in Table 2. 
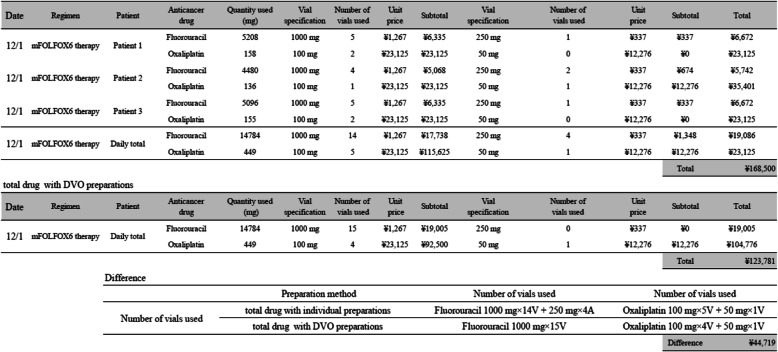

**Table 2:** Example of daily total drug cost with individual and drug vial optomization preparations

Total drug cost with individual preparations													
**Date**	**Regimen**	**Patient**	**Anticancer drug**	**Quantity used (mg)**	**Vial specification**	**Number of vials used**	**Unit price**	**Subtotal**	**Vial specification**	**Number of vials used**	**Unit price**	**Subtotal**	**Total**
12/1	mFOLFOX6 therapy	Patient 1	Fluorouracil	5208	1000 mg	5	¥1,267	¥6,335	250 mg	1	¥337	¥337	¥6,672
			Oxaliplatin	158	100 mg	2	¥23,125	¥23,125	50 mg	0	¥12,276	¥0	¥23,125
12/1	mFOLFOX6 therapy	Patient 2	Fluorouracil	4480	1000 mg	4	¥1,267	¥5,068	250 mg	2	¥337	¥674	¥5,742
			Oxaliplatin	136	100 mg	1	¥23,125	¥23,125	50 mg	1	¥12,276	¥12,276	¥35,401
12/1	mFOLFOX6 therapy	Patient 3	Fluorouracil	5096	1000 mg	5	¥1,267	¥6,335	250 mg	1	¥337	¥337	¥6,672
			Oxaliplatin	155	100 mg	2	¥23,125	¥23,125	50 mg	0	¥12,276	¥0	¥23,125
12/1	mFOLFOX6 therapy	Daily total	Fluorouracil	14784	1000 mg	14	¥1,267	¥17,738	250 mg	4	¥337	¥1,348	¥19,086
			Oxaliplatin	449	100 mg	5	¥23,125	¥115,625	50 mg	1	¥12,276	¥12,276	¥23,125
												Total	¥168,500
**Total drug cost with DVO preparations**													
**Date**	**Regimen**	**Patient**	**Anticancer drug**	**Quantity used (mg)**	**Vial specification**	**Number of vials used**	**Unit price**	**Subtotal**	**Vial specification**	**Number of vials used**	**Unit price**	**Subtotal**	**Total**
12/1	mFOLFOX6 therapy	Daily total	Fluorouracil	14784	1000 mg	15	¥1,267	¥19,005	250 mg	0	¥337	¥0	¥19,005
			Oxaliplatin	449	100 mg	4	¥23,125	¥92,500	50 mg	1	¥12,276	¥12,276	¥104,776
												Total	¥123,781
		Difference											
				Preparation method				Number of vials used			Number of vials used		
		Number of vials used		total drug with individual preparations				Fluorouracil 1000 mg×14V + 250 mg×4A			Oxaliplatin 100 mg×5V + 50 mg×1V		
				total drug with DVO preparations				Fluorouracil 1000 mg×15V			Oxaliplatin 100 mg×4V + 50 mg×1V		
												Difference	¥44,719

Based on these estimates, the drug costs for individual vials and DVO preparation were US$3,305,595 and US$3,092,955, respectively, resulting in a difference of US$212,640 for the 1-month study period. Table 3 presents the differences in the total cost between individual vials and DVO preparation for the top 10 drugs.

**Table 3:** Breakdown of reduction in drug cost by individual preparation and by DVO preparation for the top 10 items by difference in amount (US $)

Ranking of cost savings	1st	2nd	3rd	4th	5th	6th	7th	8th	9th	10th
Drug name	Bevacizumab	nab-Paclitaxel	Nivolumab	Ramucirumab	Trastuzumab	Panitumumab	Oxaliplatin	Cetuximab	Irinotecan	Paclitaxel
Drug cost by individual preparation	436,858	256,228	1,036,991	253,065	172,841	185,129	99,009	97,670	22,896	42,448
Drug cost by DVO preparation	389,034	208,465	1,012,020	232,808	158,831	171,704	87,125	91,964	17,662	37,752
Cost saved	47,824	47,763	24,971	20,257	14,010	13,425	11,884	5706	5234	4696

US$1 = ¥110

*DVO* Drug vial optimization

These results revealed the annual amounts, i.e., US$39,667,140 and US$37,115,460, for individual vials and DVO preparation, respectively, with a difference of US$2,551,680.

## Discussion

The study results indicated that annual spending on all anticancer drugs in Japan could be reduced by 6.4% by implementing DVO. Based on the estimated size of the Japanese injectable anticancer drug market in 2017 of US$7,227,096,043, the annual savings would be US$464,899,573, thus representing savings of 3.7% for entire anticancer drug market [1]. This percentage cannot be neglected when considering limited the limitations on the medical resources.

Our estimation revealed that for therapy with specific cytotoxic and molecular targeted agents, which are expensive drugs used in large quantities, noticeable cost reductions would be expected. These results are consistent with the findings of other similar studies [4, 5, 10, 11]. Further, some frequently used cytotoxic agents and molecular targeted agents might account for 70% of disposal costs [10]. Recently, expensive drugs such as molecular targeted agents and immune checkpoint inhibitors have entered the market, leading to significant increases in the total drug expenditures [12–17]. The use of such drugs is expected to increase further in the future; thus, the economic impact of DVO is expected to increase accordingly.

Because of the differences in the medical insurance systems, few economic studies related to DVO have been conducted in Japan. However, a study in the United Kingdom did report that repeated use of vials showed significant cost savings, especially for molecularly-targeted drugs [11]. In this study, vials could be used repeatedly is possible for 7 days. However, our estimates were based on the number of vials needed to achieve same-day-only repeated use. Thus, the potential economic savings in Japan could exceed the estimation specified in this study. Further, two Italian investigations found that disposal costs accounted for 4.8 and 8.3% of the annual costs of anticancer drug therapy, respectively [10, 18]. These percentages are in line with our finding of 6.4%, and in terms of medical economics, we believe that solving the problem of disposal costs is a global issue.

In the medical insurance system in Japan, the presence or absence of residual liquid is not considered important by medical institutions because the patient is charged for the total amount per vial opposed to the amount of drug actually used. Hence, the implementation of DVO, which would reduce the use of medical resources, has not progressed. With Japan’s method of calculating drug costs, the implementation of DVO means that only the cost of using a closed system drug transfer device would be borne by the medical institution. Disposal costs associated with residual liquid from drug preparations in Japan will continue to increase more rapidly than observed in Europe and the United States, in which DVO is widely implemented.

Unlike the United States, Japan does not use multidose vials, and the drug packages insert only mention single-dose vials. Furthermore, until the release of the Guidance on Safe Multiple Use of Injectable Anticancer Drugs in 2018, it was mandated that vials should be punctured, used once, and quickly discarded. Large-dose products were introduced earlier in Europe and the United States than in Japan based on the premise of DVO [6]. The use of large-dose products is particularly economical for general-purpose products, and the development of an environment in Japan in which such products can be used will also be useful in reducing costs [19].

On the contrary, it was suggested in recent theoretical economic models that the use of standard oversized vials of cancer drugs that are very large to be used by a single person represents the cause of drug waste [20, 21]. To reduce the waste of anticancer drugs, special attention should be paid to giant vials, and consideration should be given to manufacturing more suitable vials when necessary.

A limitation of this study was that the cost estimation was performed using data collected from a single facility, although it was a leading cancer hospital in Japan. In addition, the drugs used and patients’ backgrounds were not considered. Additionally, because the annual figure was estimated using data for a single month, the data may not strictly reflect the annual figure.

## Conclusions

According to our estimates, the implementation of DVO in Japan could lead to potential annual cost savings of US$464,899,573. Currently, DVO has not been promoted because of the lack of incentives in the Japanese medical insurance claims system. However, given the sizable amount of medical waste, institutional reform as a national policy is urgently needed.

## Data Availability

The data supporting the findings of this study can be made available by the corresponding author upon reasonable request.

## Abbreviation

DVO: Drug vial optimization
